# As to Influenza

**Published:** 1918-12

**Authors:** George F. Cott

**Affiliations:** Prof. Oto-Laryngology, University of Buffalo, N. Y.


### ORIGINAL ARTICLES

### As To Influenza.

GEORGE F. COTT, M. D.,

Prof. Oto-Laryngology, University of Buffalo.

The present epidemic of Spanish Influenza recalls the great pandemic about 1889 followed by several minor ones since, during which the civilized world was swept quite thoroughly. Millions of cases occurred like magic in our own country. The disease ostensively had its origin in Russia and spread from ocean to ocean in a very short time. At that time the influenza bacillus was found to have been the source of the infection. Thousands of lives were sacrificed while most of the vast army of the afflicted recovered. But did they recover? Many undoubtedly did, but the greater number never regained normality. All those who apparently recovered would pass muster by the average physician, however, when closely examined it was found that the accessory cavities of the nose were still in a receptive mood; most of them, during certain seasons of the year, were subject to repeated "colds."

That great epidemic produced its fatal effect by way of pneumonia as does this present one, and the second result was meningitis.

The present epidemic may or may not be due to the bacillus influenza. In one camp the bacillus is found. In another camp the microccus catarrhalis is the predominating germ, among others the pneumococcus is found. Whatever the cause the fatalities have been numerous. Some doctors find fatal issue only by way of pneumonia; others, toxemia, again others, both conditions prevail.

The infection makes its entry through the nose and mouth, that is commonly accepted, but it is not always that nasal symptoms are present. At first more often there is sore throat, however, great prostration is present in severe cases, leaving its effect long after apparent convalescence. In 1895 there were many cases of meningitis, always fatal, few of which have appeared at the present time. Neuritis of the cranial nerves with active suppurative sinusitis was common. Patients suffered intense agony. Whether the disease will cause chronic catarrhal quiescent or latent sinusitis this fall, remains to be seen.

Before 1889 very little had been written about sinus disease; partly because comparatively few cases existed and partly because there was no special reason for studying those cavities so generally. Since then, however, so many cases presented themselves that the literature is teeming with the anatomy, probable physiology, symptoms and treatment of sinus affections. I believe that in most cases of the Spanish Influenza that have recovered the sinuses will be found crippled, although many no doubt recover entirely. Physicians as a rule do not admit that the different sinuses of the skull are a causative factor, or even are involved, when certain symptoms exist which seemingly have no relation either immediate or remote to those cavities.

As early as 1911 I called attention to affections of the sinuses as factors in producing pain without any other symptoms, especially intracranially, which resisted all manner of treatment. These sufferers may never have had any discharge from the nose or down the throat, at least they never remembered it if they had, yet in most instances I could gradually by close questioning get them to recall having had a severe cold with profuse thick discharge from the nose. The rule is that the first attack of a ''cold'' causes prostration with headache or pain either mild or very severe, referred often back in the eyes, over the forehead, back of the head, over the temples (not the isolated temple pain spoken of by Patrick as indurative headache) or pain in the nose, accompanied by this discharge, after a few days becoming thick. All the symptoms may improve and recovery take place. The sensitive lining of the sinuses however does not usually resolve into its former status, but remains hypersusceptible. And from time to time symptoms recur in the shape of a ''cold'' which usually lasts several weeks. It is in these patients where a slow toxemia is often found. When such a patient presents himself looking anything but healthy, blood pressure may be found low (I have observed it to be as low as 90 systolic) and temperature 97 to 97½. They say they feel sick, no ambition, weak after slight exercise, etc. Now in what condition do we find the sinuses? Usually when there is no discharge, no apparent change is found in the mucous membrane in the nose, no bony or soft obstruction, no polypi, X-ray shows nothing; we conclude that there is no sinus disease. That is just where we make our mistake. All of these symptoms may be, and often are, absent. When a patient presents himself with either one of the following symptoms the sinuses are examined: intense headache, severe pain in the nose, eyes or forehead, over frontal region, in center of brain as they describe it, back of the head, or occipital region. Intense harassing cough especially at night; or several of these symptoms at one time, or complains of severe successive colds in and out of season, recurrent laryngotracheitis with cough, raising only small pieces of semi-solid secretion; or, where these symptoms occur periodically, the sinuses are suspected. Then the process of elimination begins. If a female, is there uterine trouble? The urine is examined for sugar, albumen and casts, blood count taken, a Wasserman made, teeth and gums as well as tonsils are scrutinized. I will eliminate hyperplasia of the mucous membrane of the sinuses, pus or polypi, for these at once direct our attention to the cavities; in the vast majority of the cases they are absent. Now if any abnormality in the nose is found as pressure of the middle turbinated bone on the septum, deviated septum, large inferior hypertrophy or simple intumescence, they should first be corrected but if they are not present, and the eyes examined and found normal, the region above the middle turbinate is cocainized and adrenalin applied (if no contrary indication). After five minutes a probe is introduced, which must be thin enough to pass between the turbinate and the septum; if the ethmoid is affected the probe will usually strike denuded bone. The frontal region may be likewise treated for this condition is mostly found where a large area is involved. After having applied this test, a number of times I found it necessary to open the frontal sinuses, the ethmoid cells or the sphenoid cavity. There was either no lining membrane present or it was found in a limited area only, especially in the frontal and maxillary sinuses. Sometimes part of the membrane was markedly thickened, or greater degeneration had taken place and the remainder of the bone was entirely denuded of its covering. Very often the symptoms are not accompanied by any discharge at all; the bone wherever touched seems dry and extremely hard as though having undergone a process of sclerosis. In fact the bone at times is so hard that the cells can hardly be broken down by the ordinary methods. The different sinuses may all be involved, but often the symptoms can be alleviated or remedied altogether by directing attention to one set, or to one side of the nose only. The most difficult cases to relieve are those with continuous discharge of pus or muco-pus without any other symptoms. Not now considering the major or radical operations, those patients often recover completely by living in a dry climate and high altitude. Damp cold weather always augments the trouble. The toxic varieties are often the most troublesome. Many of these cases recover completely by drainage, while others, partially only; but in all, the symptoms are markedly modified.

Why are the accessory sinuses so markedly and permenently affected while the rest of the nasal chambers seemingly recover so quickly? The mucous membrane of the nose and accessory cavities has a peculiar formation due to its varied nerve supply. This consists of secretomotor, vasodilator and vasoconstrictor, and sensation from the trifacial; all these nerves of special sense receive impulses not only from the mucous membrane of the nose but also from the skin which is demonstrated when sitting in a draft causing one to sneeze. "The mucous membrane is thick and spongy containing many glands some of which secrete a fluid rich in mucus while others secrete a fluid containing albumin and hence fall into the two catagories of mucous and serous glands." (Brubaker).

The function of the nose is to secrete thin fluid according to needs and thereby warm the air and moisten it. In addition all the air needed to carry oxygen passes through the nose, which is sifted and strained anteriorly by the vibrissae.

The fourth function of the nose, nowhere else stated, is drainage, which nature has made necessary because the higher civilization now necessitates the use of a kerchief which lower animals so far have refused to adopt. The mucous membrane of the nose has more resisting power than that of the sinuses. They are hidden or closed in a space hollowed out of or formed by thin plates of compact bone situated between the nasal cavities and the orbit, except the frontal and sphenoid sinuses and the antra. All of these open into the nasal chamber by one or several pinhole foramina, located not at the most dependent point but higher up so that drainage can only be had by capillary attraction. The mucous membrane of the nose must needs be firm and resistant to withstand pressure of more than fifteen pounds to the square inch with every effort at respiration. Not so that of the sinuses. For that reason the membrane of the nose often resists invasion of virulent bacteria which readily affects the contiguous sinuses and less often the throat, larynx and bronchi. This sensitive condition of the lining membrane of these accessory cavities is the direct cause of destruction of it in part or whole followed by all the dire symptoms alluded to above.

Now every case following influenza must be estimated according to the resisting power of the individual. Pathological conditions may be similar; the effect produced, however, quite different. While some need complete drainage others require opening of one or two places only. While some need drastic measures others may be treated symptomatically or palliatively without breaking continuity of tissue. In fact the sinus treatment depends upon the severity of symptoms, palliative, focal drainage, separate cell opening, regional surgery or radical methods, either on one side or both. Often the further procedure depends upon discovery while under treatment. I have gone into detail somewhat, in this matter because of such diversified opinions. In the matter of acute or chronic sinusitis with discharge of pus there is but one way to treat them; but in the latent variety treatment varies, not with conditions, but symptoms.

It would seem that patients who have old or chronic exudative or quiescent sinusitis would be more susceptible to the influenza bacillus or other virulent germs during an epidemic, but such seems not to be the case. During the present epidemic I have not heard of a single case having contracted the disease so far, they are quite immune. Now how do these cases terminate, with or without treatment. Those who have pain, usually recover by drainage; when there is discharge and drainage is not inhibited the patient is exposed to all sorts of infection. I have had six cases of fatal meningitis, two developed tuberculosis. Those operated for pneumatic drainage, and a number of muco-purulent cases becoming dry afterwards, seems as numerous as the ordinary healthy individual and just as comfortable

After the present epidemic has subsided it may be well to look out for the following, perhaps all toxic symptoms, if not for treatment, at least, for the purpose of accurate diagnosis passive or continuous abdominal and gastric pains, gradually appearing deafness, chronic pasty pharyngitis, severe and often persistant cough, pain along branches of the sensory nerves as the trifacial, pneumogastric, especially its laryngeal and ottic branches and the glossopharyngeal. Severe and most distressing headache, pain in the eyes, usually deep-seated, partial blindness, scotoma, severe dizziness, apoplectiform seizures, etc. Of course all these conditions may be caused by toxemia from other diseases but often isolating the sinuses and no other cause can be found, treatment directed to the skull would usually prove effective.

Climate has a powerful influence on those suffering with mucopurulent discharge, or pus with rather a small number of cocci. I found by removing such patients to high altitudes where the climate is dry they recover promptly and permanently. The same effect is produced in those patients afflicted with the condition which we call catarrhal deafness where the internal ear is not involved.

In this connection it is worth mentioning that children suffer equally with adults. We were taught, as will be found in Gray's anatomy that the several accessory cavities of the nose develop from birth to puberty and therefore as they are so primitive the influenza bacilli would find them immune. We now know that these sinuses begin in early fetal life and are fairly well developed in infancy so that the children in arms as well as kindergarten and grade pupils are liable to this infection. This is important, however, that the child before puberty does not suffer so disastrously as the adult, either in fatality or permanent injury.

The present epidemic, so far not nearly as disastrous as that of '89 and the second one of 1894 and 1895, will leave much suffering in its wake long after the epidemic has been forgotten and most of it will be found located in the sinuses of the skull accompanied by a train of symptoms, differing in each patient. In acute exacerbation there is sometimes discharge accompanying the ''cold'' which varies in consistency from profuse watery to thick pus. The examination of the secretion may show streptococci, straphylococci, pneumococci, sometimes isolated bacilli; but rarely the influenza bacillus, often pus cells without any cocci.

Doubt as to the specific cause is as great as ever. That the several isolated or mixed bacteria, which may include influenza bacilli have assumed a highly virulent type seems probable.

But the cause of this assumed periodical virulency can never be adequately explained. Any one of them, usually mildly active, may at a time like this become highly toxic. This is shown by the findings in different parts of the country by the bacteriologists. For this reason it is so difficult to procure antitoxin which will successfully combat the disease, as in diphtheria.

Our sanitary officers deserve great credit in limiting the spread of the contagion to but a limited number of cases, in camp with many thousands of soldiers as well as in cities. In Buffalo particularly it was masterfully handled and all due to the power given to Dr. Gram to enforce sanitary laws strictly without fear or favor. I wonder whether the average citizen appreciates the efforts of our energetic and most efficient Health Commissioner.

It would seem that many patients die, but if you consider the large number of cases, you will see that the mortality is really quite low. One physician of my acquaintance treated over 700 cases recently. He encountered but four cases of pneumonia, one an asthmatic patient who died and another who had some tubercular bone lesion previously. All the others recovered, save one, a convalescent, who succumbed suddenly with acute dilation of the heart on the third day after having left her bed.

Whether that percentage will continue throughout the epidemic or not I cannot say at present. He met with primary symptoms such as hemorrhage of the bowels, also of the throat and nose. Fifty cases had severe abdominal pain and almost all had severe pain in the back.

Let me cite but a few cases exemplifying principally the latent variety of sinus disease which had never been suspected.

Case 1. Mrs. M., age 56. About thirty years ago suffered a great deal of pain over the eyes and temples anteriorly during the whole winter for six or seven years. There was so much pain in eyes that she continually changed glasses. On examination by a laryngologist nothing was found. Never had any extensive colds or much discharge. Lately some watery discharge in morning. Also had pain in right eye following an attack of grippe she says. The patient would pass for 65 so careworn and wrinkled from suffering. After ascertaining the extent of her troubles both sides were operated upon at the same time. She improved rapidly and now after three years she considers herself well and says she is most thankful.

Case 2. Female, age 29, married, no children. Severe pain back of head at base almost intolerable causing intense suffering. Pain was of a stabbing nature. Sometimes eyes blurred and at other times she couldn't see at all. The pain back of head caused much depression. Temperature normal, well nourished.

Upon examination I found she had an old ethmoid trouble which however seemed to be quiescent, no discharge nor any other indication of nasal disease. In this case I hesitated to operate because of this previous history: two years ago she had a tumor of the left ovary removed at the Gratwick Laboratory reported as carcinoma. When she began with the present pain and eye symptoms, she went to the same surgeon who found she had beginning chocked disc and concluded that she probably had a brain tumor of a cancerous nature, metastasis. He referred her to an ophthalmologist who also found the beginning chocked disc. She then saw a neurologist who corroborated the first diagnosis. She then called on a laryngologist who after having examined the nose said he could find nothing abnormal.

With such a history I could hardly be blamed for hesitating to operate on the ethmoid cells. However after four weeks treatment she was getting worse and insisted on having something done whatever the consequences. I then cleaned out the ethmoid cells under cocaine anesthesia on one side only in my office. In less than a week her symptoms had all disappeared. After some weeks I met the neurologist, related the case and he then told me that such cases often acted that way and in about six months the symptoms would appear. It is now two years and the patient is apparently perfectly well.

Case 3. A man aged 63, stocky and well built. One and one-half years ago while carrying a satchel in New York suddenly felt his hands get numb and some headache with double vision. After a few days he had completely recovered. In a second attack about ten months later he lost control of his right hand. Remained in bed for a week but recovered the use of his hand again in three days. Eight months later he had a third attack, arm became lame and mouth twitched for three days when the symptoms again wore off He was under the observation of a most competent physician who could not account for those symptoms of paralysis. He asked me to see the patient. He seemed in good physical condition, urine normal, blood pressure 140 systolic but he had an old ethmoiditis. He is now entirely well but will be kept under observation for some years.

Cases 4 and 5 were similar, both females, one aged 18 the other 38. Both developed a cellulitis, in the older one the region of the lower jaw and neck was involved, in the younger one the entire lateral neck on one side so that she could not move her head at all. Both recovered after several weeks. Both had chronic ethmoiditis.

The symptoms in the above cases were due to toxemia due to the ethmoid disease of which there was no previous history that the patients remembered. Yet no doubt these patients passed through a mild attack of one of the numerous pandemic, epidemic or endemic influenza which we observed since 1889 or they had it some time in suppurative pansinusitis, for otherwise so much destruction of tissue covering the cells could not have taken place. If no other cause can be assigned for many of the obscure symptoms causing so much pain and suffering it is well always to examine the ethmoid region or other of the accessory cavities.

1001 Main St.

---

**Chronic Appendicitis With Pseudomyxomatous Diverticulum.** Villar, Le Prog. Med., Mch. 30, reports a case in a man of 46. The appendix was enlarged and had an extra-appendicular pocket filled with gelatinous material.

---

**Spinal Anaesthesia in War Surgery.** Riche, Le Prog. Med., Mch. 30, employed spinal anaesthesia in 2200 cases between 1908 and 1918; Desplats 550. Thus consider the increase of shock a myth. They employ stovaine. For 5 c.g., 3 are eliminated in 2 hours, 1 in the third hour, ½ in the fourth.

